# An Early Catch: Multisystem Inflammatory Syndrome in Adults (MIS-A) in a Young Adult Male With COVID-19 Exposure

**DOI:** 10.7759/cureus.26519

**Published:** 2022-07-02

**Authors:** Farzam Khokhar, Zainab Bhura, Muhammad Muneeb, Garima Singh, Jianghong Yu

**Affiliations:** 1 Internal Medicine, State University of New York Upstate Medical University, Syracuse, USA; 2 Internal Medicine, University Health Network, Toronto, CAN; 3 Internal Medicine, Ziauddin University, Karachi, PAK; 4 Internal Medicine, Saint Vincent Hospital, Worcester, USA; 5 Rheumatology, State University of New York Upstate Medical University, Syracuse, USA

**Keywords:** intravenous immune globulin, internal medicine and rheumatology, academic rheumatology, pulmonary critical care, vv ecmo, sars-cov-2, covid 19, multisystem inflammatory syndrome, multisystem inflammatory syndrome in children, multisystem inflammatory syndrome in adult

## Abstract

While severe acute respiratory syndrome (SARS) is the most common presentation of coronavirus disease 2019 (COVID-19) infection, several short- and long-term complications from COVID-19 infection are also being recognized. One such complication with life-threatening consequences is known as multisystem inflammatory syndrome in adults (MIS-A). While the phenomenon of multisystem inflammatory syndrome in children (MIS-C) is more recognized, the pathophysiology of both presentations remains a mystery currently. Several theories have been put forward however no consensus has been established yet. We present the case of a 20-year-old male who was admitted to the intensive care unit for a multisystem illness characterized by severe biventricular failure, profound shock, and acute liver and kidney injuries. The severity of illness necessitated the treatment with mechanical ventilation, extracorporeal membrane oxygenation (ECMO), vasopressors, and continuous veno-venous hemofiltration (CVVH). The patient was treated with one dose of intravenous immune globulin (IVIG). In association with the foregoing treatment, the patient made dramatic recovery and came off pulmonary, hemodynamic, and renal support within a week and made remarkably quick and full recovery. This case highlights a rare presentation of a COVID-19 complication that requires prompt recognition, supportive care, and empiric treatment that led to a favorable outcome in this case.

## Introduction

The rapidly developing coronavirus was first identified in Wuhan, China, in December 2019. Since then, over 530 million cases have been confirmed, and over six million deaths recorded [1]. Previously, it was believed that children were resilient to this virus. However, by mid-May 2020, the World Health Organization (WHO) published a statement regarding children and adolescents requiring admission to the ICU following the coronavirus disease 2019 (COVID-19) infection. Children were believed to have a multisystem inflammatory condition with features similar to Kawasaki disease and toxic shock syndrome [2]. They manifested fever, rash, non-purulent conjunctivitis, coagulopathy, gastrointestinal symptoms, and peripheral edema, with elevated inflammatory biomarkers [3]. The Royal College of Pediatrics and Child Health (RCPCH) labeled this condition as pediatric multisystem inflammatory syndrome temporally associated with COVID-19 (PIMS-TS) [4]. As cases continued to rise, the Centers for Disease Control and Prevention (CDC) and the WHO tagged this illness as a multisystem inflammatory syndrome in children (MIS-C) [5].

A parallel syndrome in adults has been similarly identified and named multisystem inflammatory syndrome in adults (MIS-A). Following the COVID-19 infection, most adults experience fever, cough, and shortness of breath. A few patients may have worsening symptoms, however, a smaller proportion of these individuals may present with multiorgan system failure. Similar to our patient, this is believed to be due to a cytokine storm [6]. This phenomenon is due to a delayed dysregulated release of cytokines, with the loss of regulatory control of proinflammatory cytokines, like interleukin-6 (IL-6), leading to widespread inflammation and multiorgan failure [7].

## Case presentation

A 20-year-old African-American male patient with no known medical history presented to the emergency department at a tertiary care center in the United States Northeast region with complaints of dull headache, sore throat, maculopapular rash on torso, feeling of warmth, and abdominal pain for four days. The patient denied any chills, rigors, body aches, constipation, nausea, vomiting, diarrhea, shortness of breath, cough, congestion, vision changes, and/or neck stiffness. At the time of presentation, the patient was alert, oriented, and fully participated in the interview. The patient chose to come to the emergency department as he had one loose, bloody bowel movement on the day of admission. Of note, the patient did arrive at the emergency department in his own vehicle.

On arrival, vitals were significant for heart rate of 135 beats per minute, temperature of 38.4°C, respiratory rate of 23 breaths per minute, and blood pressure of 97/70 mmHg. Physical examination was significant for dry mucous membranes, tachycardia, generalized, non-focal "discomfort" on abdominal palpation, no frank blood on rectal examination, coalesced maculopapular rash covering at least 50% of the patient's anterior trunk, not present as much on posterior trunk, sparing the face, non-pruritic and not tender.

Sepsis protocol was promptly initiated after initial assessment which included 30 cc/kg intravenous fluid resuscitation, broad-spectrum antibiotic coverage with vancomycin, and piperacillin/tazobactam. Additionally, two sets of blood cultures, urinalysis, sputum cultures, lactic acid, and community-acquired diarrhea panel were also sent out (it should be noted here that other than elevated lactic acid, the rest of the sepsis workup returned back negative). Tachycardia mildly improved after fluid administration, however, blood pressure continued to drop and the patient was started on norepinephrine. Also, the patient's respiratory status deteriorated and he went from being on room air to requiring 3 L nasal cannula, to 15 L oxygen mask, and to high flow nasal cannula, all within a matter of an hour. Subjectively, the patient was still alert and oriented and able to speak, however, in shorter sentences and stated he felt it is difficult for him to exhale. Chest x-ray obtained in the ED was normal. Computed tomography (CT) imaging was not able to be obtained in the acute setting as the patient was deemed too unstable at the time of presentation. COVID polymerase chain reaction (PCR) test was negative at the time of admission. Initial presumed diagnosis at the time of admission was septic shock. Soon after, the patient was transferred to the medical intensive care unit (ICU). Laboratory results are shown in Table 1.

**Table 1:** Relevant laboratory data from the hospitalization. ANA: antinuclear antibody

Variables	On admission	Reference ranges
White cell count (per μL)	8,400	4,500-13,000
Hemoglobin (g/dL)	14.7	13.5-18.0
Hematocrit (%)	45.1	41-53
Platelet count (per μL)	79,000	150,000-400,000
Creatinine (mg/dL)	2.3	0.70-1.20
Alanine aminotransferase (U/L)	1,906	<41.0
Aspartate aminotransferase (U/L)	5146	<40.0
Lactic acid (mmol/L)	4.0	0.50-2.2
C-reactive protein (mg/L)	215.9	<8.0
Erythrocyte sedimentation rate (mm/h)	17	<15.0
Ferritin (μg/L)	11,826	30-400
D-dimer (μg/mL)	>20.00	<0.50
Total bilirubin (mg/dL)	2.7	<1.20
Alkaline phosphatase (U/L)	83	40-129
Protime (s)	46.1	12.5-14.9
International normalized ratio	4.79	~1.0
ANA, nucleolar pattern	<80	<80
C3-complement (mg/dL)	150	90-180
C4-complement (mg/dL)	33	10-40
Anti-myeloperoxidase Ab (CU)	<5.0	<20.0
Anti-proteinase 3 Ab (CU)	<5.0	<20.0

Within hours of admission to the ICU, the patient’s hemodynamic compromise and respiratory status continued to deteriorate. After failing trials of non-invasive positive pressure ventilation, he required intubation and mechanical ventilation. Vasopressor requirements continued to increase, and the patient was hypotensive even on four vasopressors. A bedside echocardiogram (ECHO) revealed severely reduced systolic left ventricular function with left ventricular ejection fraction (LVEF) of 15% as can be seen in Video 1. Right ventricular systolic function was also reported to be reduced.

**Video 1:** Echocardiogram showing visually estimated left ventricular ejection fraction of approximately 15%.

At this time, it was decided to initiate extracorporeal membrane oxygenation (ECMO). In the interim, the patient’s serum creatinine continued to rise to a maximum of 2.87 mg/dL and he was also noted to be anuric. Nephrology initiated the patient on continuous renal replacement therapy (CRRT). At the same time, he was also noted to have shock liver with severe transaminitis as can be seen in Table 1.

Upon further testing and collateral information from the family, it was brought to our knowledge that the patient had experienced symptoms of anosmia and ageusia approximately a month prior to admission. Notably, the patient was never vaccinated against COVID-19. This prompted us to test for COVID-19 IgG antibodies which indeed did come back positive. No laboratory test for COVID-19 IgM antibodies was available. At this time concern for multisystem inflammatory syndrome in adults (MIS-A) was brought forward by the rheumatology consult service. As per their recommendation, the patient received one-time dose of 1 g/kg (total ~80 g) of intravenous immune globulin (IVIG) approximately 48-54 hours from initial presentation to the emergency department. Approximately 18-20 hours after the dose of IVIG, the patient was started on dexamethasone 10 mg/BSA m^2^ daily for weeks one and two, 5 mg/BSA m^2^ daily for weeks three and four, 2.5 mg/BSA m^2^ daily for weeks five and six, and 1.25 mg/BSA m^2^ daily for week seven and taper dose to zero in week eight.

Remarkably, the patient made rapid recovery within 24 hours of IVIG and 4-6 hours of dexamethasone administration. His vasopressor requirements started to come down, urine output picked up and ventilatory requirements improved. Over the next 48-72 hours he was taken off all pulmonary, hemodynamic, and renal support. Additionally, significant improvement in laboratory markers was seen namely in ferritin, c-reactive protein (CRP), lactic acid, and transaminases. Blood cultures, urine cultures, sputum cultures, and stool diarrhea panel did not report any positive results. A repeat echocardiogram performed approximately three days after the original one showed an improvement in left ventricular ejection fraction to 50% as can be seen in Video 2.

**Video 2:** Echocardiogram showing visually estimated left ventricular ejection fraction of approximately 50%.

The patient had a prolonged hospitalization of approximately three weeks as the severity of his illness necessitated a prolonged recovery which included intense inpatient physical rehabilitation. He has been followed closely outpatient and was seen most recently approximately 13 months from his initial presentation and is back to work and believes his physical function has returned to baseline.

## Discussion

As a newly emerged clinical syndrome, MIS-A is a poorly understood and a rare complication of COVID-19, with only 20 patients meeting the Centers for Disease Control and Prevention (CDC) criteria of MIS-A between December 2020 and April 2021 in the United States of America [8]. Due to the uniqueness of this disease, multiple studies have emerged within the last two years to identify the correlation between age, gender, and the treatment of this disease. According to Patel et al.’s review, MIS-A mainly occurs in young males ranging from the ages of 19 to 34 years, who are non-Hispanic Black or Hispanic persons [9]. It is believed to be characterized by abdominal pain, conjunctivitis, carditis, gastrointestinal symptoms, skin rash, and vomiting. These symptoms fall under the spectrum of Kawasaki disease and toxic shock syndrome, unlike the classic COVID-19 symptoms [8]. The first ever case series study conducted by CDC included 11 patients from March to August 2020, seven of whom on presentation were in cardiogenic shock [10].

It is noted in the study conducted by Belay et al. that patients who tested positive for SARS-CoV-2 infection have approximately one month before the onset of MIS-A [11]. It is crucial to emphasize that 57% of the patients diagnosed with MIS-A were admitted to the intensive care unit (ICU), requiring vasopressor support or hemodynamic monitoring, and 7% died [9]. Comparably, our 20-year-old patient was transferred to the ICU for further workup on the day of admission after his cardiopulmonary collapse, eventually having an ejection fraction of 15%.

It is proposed by Siddiqui and Mehra that the process of COVID-19 has the following three stages [12] - (1) early infection phase where SARS-CoV 2 replicates itself in the host, (2) pulmonary stage during which there is an inflammatory response presenting the symptoms of COVID-19, and (3) hyperinflammation stage which is formed by the hosts' inflammatory response.

It is believed that MIS-A is due to a delayed, dysregulated immune response making it a part of stage 3, the hyperinflammation phase. The influx of T-cells, inflammatory monocyte-macrophages and neutrophils, proinflammatory cytokines, and chemokines, leads the body into hyperinflammation. It is plausible that this may be due to the downregulation of the renin-angiotensin-aldosterone system, or endothelial damage and thrombo-inflammation caused by COVID-19 [9].

Due to the overactivity of the immune system, by the time the patient presents to the hospital, they have around five organ presentations, which may lead to an array of differentials [9]. However, according to Morris et al., clinicians and health departments should consider MIS-A in adults, who present with the systems aligning with the case definition provided by the CDC [10]. This should be confirmed via identifying a previous COVID-19 infection in patients through PCR or antigen test results, even if the patient had minimal or no symptoms of SARS-CoV-2 [10]. Likewise, within our case report, the patient had tested positive for COVID-19 antibodies, with minor COVID-19 symptoms a month prior. Other than identifying the presence of COVID, it is preeminent to test for CRP, ferritin, neutrophil count, ESR, and fibrinogen, which have been reported to be elevated in MIS adolescents [13].

Once confirmed via PCR, the mainstay treatment for this complication is a combination of immune modulators (e.g., corticosteroids, IVIG, anakinra) and anticoagulants. Our patient did not receive anakinra or antithrombotic therapy due to a lack of guidelines available at time that this encounter took place. Even though underlying management has been identified, it is crucial to have a multidisciplinary approach. Similarly, we constantly communicated with the rheumatology, nephrology, and infectious disease teams.

## Conclusions

Multisystem inflammatory syndrome in adults (MIS-A) is a rare but fatal complication of COVID-19 infection. Our case report highlights that while the active infection with COVID-19 may be very mild/asymptomatic and may go unnoticed, MIS-A can still present as full-blown multisystem failure requiring hemodynamic, pulmonary, and renal support. Patients can present with acute kidney injury, pulmonary injury, cardiovascular collapse, and non-pruritic, non-tender skin rash which may create an array of differentials, overlooking MIS-A for an alternative diagnosis. While the pathophysiology continues to remain controversial, what is currently agreed upon is early recognition and prompt supportive and empiric treatment with immune modulators (e.g., corticosteroids, IVIG, anakinra) and antithrombotic therapy may result in a favorable prognosis. Furthermore, the question remains if there is any correlation between MIS-A and specific age groups, gender, and ethnicity.
